# Characterization of Fosfomycin Resistance Gene, *fosB*, in Methicillin-Resistant *Staphylococcus aureus* Isolates

**DOI:** 10.1371/journal.pone.0154829

**Published:** 2016-05-04

**Authors:** Zhuyingjie Fu, Yang Liu, Chunhui Chen, Yan Guo, Ying Ma, Yang Yang, Fupin Hu, Xiaogang Xu, Minggui Wang

**Affiliations:** 1 Institute of Antibiotics, Huashan Hospital, Fudan University, Shanghai, China; 2 Key Laboratory of Clinical Pharmacology of Antibiotics, Ministry of Health, Shanghai, China; University Hospital Münster, GERMANY

## Abstract

To investigate the prevalence, location and genetic environments of fosfomycin-resistance (*fos*) genes in methicillin-resistant *Staphylococcus aureus* (MRSA) clinical strains, 67 fosfomycin-resistant MRSA strains were isolated from the blood and cerebrospinal fluid samples at a teaching hospital in Shanghai. The presence of *fos* genes in these clinical strains was detected by PCR and sequencing. The locations of *fos* genes were determined by Southern blotting and genetic environments were analyzed by primer walking sequencing. Multiple locus sequence typing (MLST) was used to characterize genetic diversity. Conjugation was performed to evaluate the transferability of *fos* genes. Among 67 fosfomycin-resistant MRSA strains, nine high level fosfomycin resistant strains (≥128 μg/ml) were *fosB*-positive. Three new subtypes of *fosB*, designated as *fosB4*, *fosB5*, and *fosB6*, were identified. *fosB1*, *fosB4* or *fosB6* genes were located on small plasmids (ca. 2.5 kb) and flanked by an analogous replication gene (*rep*). Differently, the *fosB5* gene was surrounded by a shorter *rep* gene and two copies of a transposon gene (*tnp*) that shared high identity with the IS*257*-like transposon. Four MLST types were found among the nine *fosB-*positive strains. Transconjugants with the *fosB* genes were resistant to fosfomycin with MIC 64 or 128 μg/ml. In conclusion, different subtypes and genetic environment of *fosB* genes indicate that gene heterogeneity for fosfomycin resistance in MRSA isolates.

## Introduction

Fosfomycin is a bactericidal antibiotic that was first discovered in 1969. By irreversibly interfering with the first committed step of peptidoglycan biosynthesis, fosfomycin can hinder the cell wall synthesis in both Gram-positive and Gram-negative bacteria [1]. Due to its unique mechanism, fosfomycin alone or in combination with other antibiotics is used for the treatment of nosocomial infections due to multidrug-resistant (MDR) Gram-positive and Gram-negative bacteria. [1] But fosfomycin can be inactivated through chemical modification with glutathione, L-cysteine/bacillithiol, phosphate, and H_2_O, which can be added to fosfomycin’s epoxide ring through the catalyzing of FosA, FosB, FosC, FosX and their subtypes, FosA1-4, FosB1-3, FosC1-2, FosX, FosX^cc^, respectively [2–7]. Among all plasmid-mediated resistance genes, only the *fosB* gene has been detected in Gram-positive pathogens [1]. The plasmids harboring *fosB1* sized from 2.4 kb to 4.1 kb that confer resistance to fosfomycin have been found in *Staphylococcus* spp. [8]. Chromosomal-derived *fosB2* has been found in *Bacillus anthracis* [9] and *fosB3* locating on a transferable circular intermediate has been found in *Enterococcus faecium* [3]. The goal of this study is to characterize *fosB* gene among 67 fosfomycin-resistant MRSA clinical isolates.

## Materials and Methods

### Bacterial Strains

Ninety-six MRSA clinical strains isolated from the blood or cerebrospinal fluid were collected from 2004 to 2014 at a teaching hospital [10]. Among them, 67 fosfomycin-resistant MRSA stored frozen at -70°C in L-broth with 40% glycerin for this study. *S*. *aureus* strain ATCC25923 (American Type Tissue Culture Collection, Manassas, VA, USA) was used as a recipient in the conjugation assay. *S*. *aureus* strain ATCC 29213 was used as a quality control strain in antimicrobial susceptibility testing experiments. *E*. *coli* strain V517 was used as a marker in Southern blot.

Antimicrobial Susceptibility Testing

The MIC of fosfomycin against clinical strains and transconjugants was based on the CLSI recommendation to use the agar dilution method [11]. The results were interpreted according to the 2012 EUCAST criteria [12].

### PCR Screening

DNA templates were prepared using the Tiangen extraction kit (TIANGEN, Beijing, China) and were screened for the presence of *fosA*, *fosB* and *fosC* genes by primers and PCR conditions as described previously [13]. PCR products were subjected to DNA sequencing for determine subtypes of *fos* genes.

### Genetic Environment Analysis

The plasmid DNA was extracted from *fosB* positive strains by alkaline lysis using the Plasmid Midi Kit (QIAGEN, Hilden, Germany). Primer walking sequencing was carried out to determine the sequences flanking the *fosB* genes.

### Southern Blot

After gel electrophoresis, plasmid DNA fragments were transferred to a positively charged nylon membrane (Roche, Mannheim, Germany) by a vacuum blotter model 785 (Bio-Rad, Hercules, USA). The *fosB* PCR product was used as the positive control, while plasmid extracted from *Escherichia coli* V517 was used as the marker. The membrane was hybridized with *fosB* probe mixed by *fosB1*, *fosB4*, *fosB5* and *fosB6* probes according to the manufacturer’s instructions for the DIG High Prime DNA Labeling and Detection Starter Kit (Roche, Mannheim, Germany).

### Conjugation Assay

Rifampicin-resistant mutants of *S*. *aureus* ATCC25923 were generated following overnight incubation in Brain Heart Infusion (BHI) broth containing one-half the MIC of rifampicin, as determined by agar dilution testing. Following overnight incubation at 37°C, bacteria were plated on BHI agar plate containing 10 times the MIC of rifampicin. Each mutant was streaked onto a BHI agar plate containing 200 μg/ml of rifampicin for 3 generations and a following 3 generation incubation on BHI agar plate containing 400 μg/ml of rifampicin. Then the rifampicin-resistant mutant of *S*. *aureus* ATCC25923 was used as recipient, and conjugation assay was carried out as previously described [14]. Putative transconjugants were selected on BHI plates containing fosfomycin (10 μg/ml), Glucose-6-phosphate (25 μg/ml) and additional rifampicin (400 μg/ml). The transconjugants with the same *fosB* subtype from corresponding donors were confirmed by Multiple Locus Sequence Typing (MLST). The MLST type of real transconjugants were same as the type of *S*. *Aureus* ATCC25923, and different from the donor strains.

### Multiple Locus Sequence Typing

Isolates were screened using a previously described method [15] to detect the following seven housekeeping genes: *arcC*, *aroE*, *glp*, *gmk*, *pta*, *tpi*, and *yqiL*. The sequences of the PCR products were compared to the existing sequences available from the MLST website (http://www.mlst.net) for *S*. *aureus* [16], and the allelic number was determined for each sequence.

Nucleotide Sequence Accession Numbers

The GenBank/EMBL/DDBJ accession number for the sequences of *fosB4*, *fosB5* and *fosB6* genes are KR870311, KT032253 and KR870314, respectively.

## Results

### Antimicrobial Susceptibility Testing and *fos* Gene Detection

The MIC of fosfomycin for the 67 MRSA strains ranged from 64 μg/ml to >256 μg/ml. Nine isolates with MIC ≥128 μg/ml were positive for *fosB* (Table 1), and no isolates were positive for *fosA* or *fosC*.

**Table 1:** Characteristics of *fosB*-positive isolates and transconjugants.

Strains	*fosB* subtypes	Fosfomycin MIC (μg/ml)	Source	Ward	Fosfomycin exposure*	Plasmid size (kb)	MLST type
SA0406	*fosB4*	>256	Blood	Dermatology	Existent	2.6	ST5
SA0409	*fosB4*	>256	Blood	Dermatology	Existent	2.3	ST5
SA0516	*fosB4*	>256	Blood	Dermatology	Existent	2.3	ST5
SA0849	*fosB4*	>256	Blood	Hematology	Nonexistent	2.3	ST5
SA1057	*fosB4*	>256	Blood	Infection Department	Nonexistent	2.6	ST764
SA1159	*fosB1*	>256	Blood	Geriatrics	Nonexistent	2.6	ST2590
SA1278	*fosB6*	>256	Spiral fluid	Neurosurgery	Nonexistent	2.9	ST5
SA1280	*fosB5*	>256	Spiral fluid	Neurosurgery	Existent	Unclear^#^	ST5
SA0620	*fosB5*	128	Blood	Neurology	Existent	Unclear^#^	ST239
Transconjugant0406	*fosB4*	64	NA^§^	NA^§^	NA^§^	2.6	NA^§^
Transconjugant0409	*fosB4*	64	NA^§^	NA^§^	NA^§^	2.3	NA^§^
Transconjugant0516	*fosB4*	64	NA^§^	NA^§^	NA^§^	2.3	NA^§^
Transconjugant0849	*fosB4*	64	NA^§^	NA^§^	NA^§^	2.3	NA^§^
Transconjugant1057	*fosB4*	64	NA^§^	NA^§^	NA^§^	2.6	NA^§^
Transconjugant1159	*fosB1*	64	NA^§^	NA^§^	NA^§^	2.6	NA^§^
Transconjugant1278	*fosB6*	64	NA^§^	NA^§^	NA^§^	2.9	NA^§^
Transconjugant1280	*fosB5*	128	NA^§^	NA^§^	NA^§^	Unclear^#^	NA^§^
Transconjugant0620	*fosB5*	128	NA^§^	NA^§^	NA^§^	Unclear^#^	NA^§^
*S*. *aureus* ATCC25923	NA^§^	2	NA^§^	NA^§^	NA^§^	NA^§^	NA^§^

*Exposure to fosfomycin within one month was defined as “Existent” history prior to the positive blood/CSF culture.

^#^Unclear = uncertainty about the location of *fosB*.

^§^NA = not applicable

### Analysis of the *fosB* Gene and Genetic Environment

**Fig 1:**
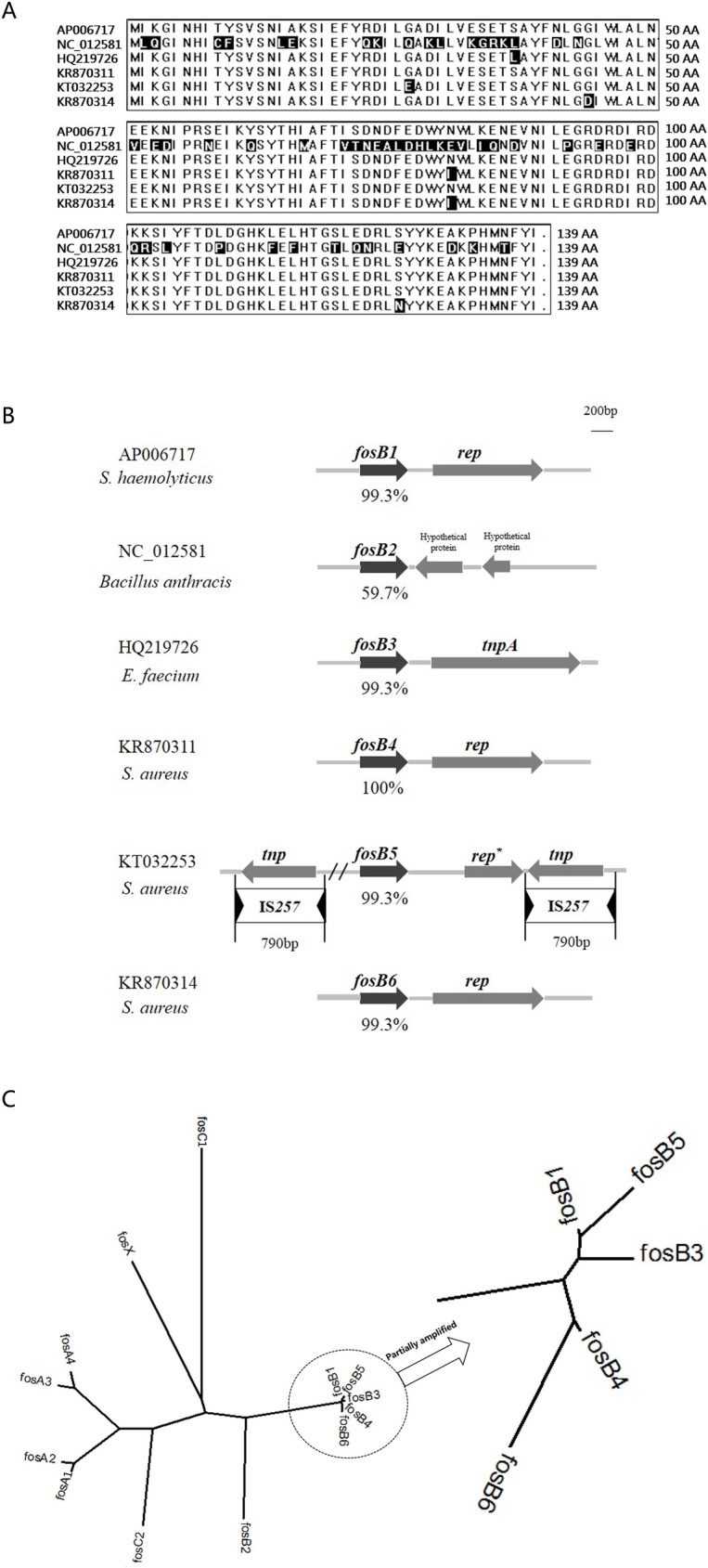
Comparison of the sequences of *fosB* genes. Diversity of amino acids among *fosB* gene subtypes (A). Diversity of deduced amino acid sequences of *fosB* and genetic environment (B). Amino acid relationships of FosB4-6 with FosA1-4, FosB1-3, FosC1-2 and FosX (C). Adjacent sequences of *fosB1-6*: *fosB1* (AP006717) in *S*. *haemolyticus*, *fosB2* (NC_012581) from *Bacillus anthracis*, *fosB3* (HQ219726) from *E*. *faecium*, *fosB4* (KR870311) from *S*. *aureus* SA0406, *fosB5* (KT032253) from *S*. *aureus* SA1280, and *fosB6* (KR870314) from *S*. *aureus* SA1278. The residues differing from the consensus sequence are boxed in inverse color. Two IS*257* elements (790 bp) are indicated as open boxes. Arrowheads within the IS*257* elements represent terminal inverted repeats. rep* means a shorter *rep* gene in SA1280, with 38.5% nucleotide identity to those in *S*. *haemolyticus* (AP006717) and *S*. *aureus* (KR870311, KR870314) which had an high identity in *rep*. The unrooted dendrogram was generated by Clustal W (http://www.genome.jp/). Proteins not mentioned above(GenBank accession no.): FosA (AAA98399); FosA2 (ACC85616); FosA3 (BAJ10054); FosA4 (BAP18892.1); FosC (CAA83855); FosC2 (BAJ10053); FosX (YP_005926752).

The *fosB* genes found in *S*. *aureus* SA0406, SA1280 and SA1278 were different in nucleotide identity and deduced amino acid sequence from *fosB1* genes discovered in plasmids from *Staphylococcus* spp. [17–18], chromosomal-derived *fosB2* genes found in *Bacillus anthracis* [9] and the *fosB3* gene from *E*. *faecium* [3] (Table 2 and Fig 1). Consequently, the *fosB* genes from *S*. *aureus* SA0406, SA1280 and SA1278 were designated as *fosB4*, *fosB5* and *fosB6*, respectively. These three *fosB* genes differing from each other by 2−4 amino acids were all 420 bp in length and encoded a 139-amino acid protein. The strains *S*. *aureus* SA0409, *S*. *aureus* SA0516, *S*. *aureus* SA0849, *S*. *aureus* SA1057, and *S*. *aureus* SA0406 shared the same *fosB4* gene. *S*. *aureus* SA0620 carried the same *fosB5* gene as *S*. *aureus* SA1280. And *S*. *aureus* SA1159 carried a *fosB1* identical to *S*. *haemolyticus* [16]. As it turns out, the *fosB4-6* genes shared a high homology (≥97.1%) with *fosB1* and *fosB3* (Table 2).

**Table 2:** Percent identity in nucleotide and deduced amino acid sequence for *fosB* subtypes.

	Nucleotide sequence			Deduced amino acid		
New subtype	*fosB1*	*fosB2*	*fosB3*	*fosB1*	*fosB2*	*fosB3*
*fosB4*	99.5%	62.2%	99.3%	99.3%	59.0%	98.6%
*fosB5*	99.8%	62.2%	99.5%	99.3%	59.0%	98.6%
*fosB6*	99.3%	60.5%	99.0%	97.8%	58.3%	97.1%

Primer walking sequencing determined that the *fosB* genes except *fosB5* were located on 2.5 kb-sized plasmids and flanked by an analogous *replication* (*rep*) gene. The *fosB5* gene located in a unique genetic environment and was surrounded by a shorter *rep* gene and two copies of a *transposon* (*tnp*) gene that shared high identity with the IS*257*-like transposon (Fig 1).

**Fig 2:**
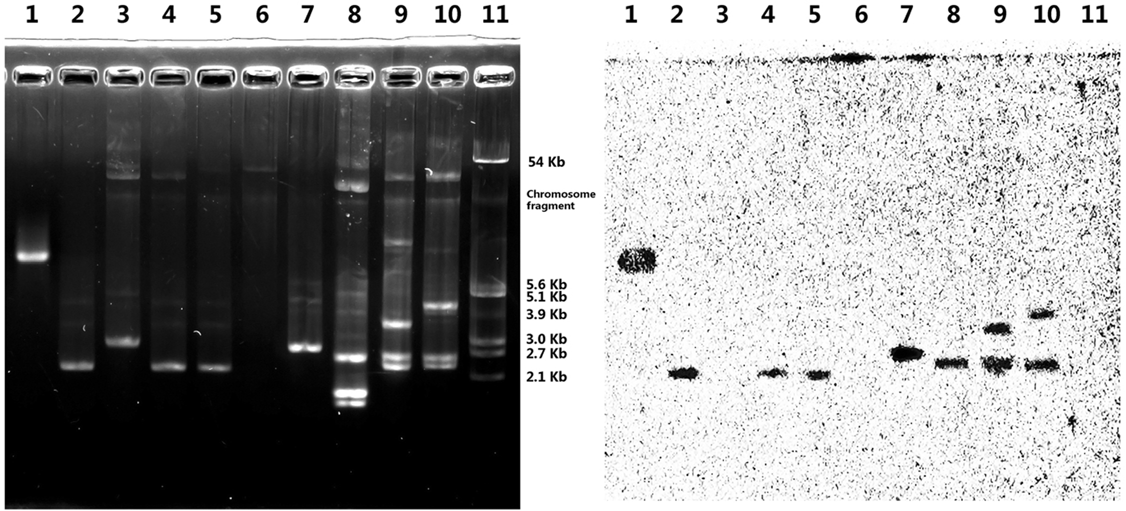
Southern hybridization analysis of *fosB*-positive MRSA isolates. Lanes: 1, PCR product of *fosB* gene as the positive control; 2, *S*. *aureus* SA0849; 3, *S*. *aureus* SA0620; 4, *S*. *aureus* SA0516; 5, *S*.*aureus* SA0409; 6, *S*. *aureus*SA1280; 7, *S*. *aureus* SA1278; 8, *S*. *aureus* SA0406; 9, *S*. *aureus* SA1159; 10, *S*. *aureus* SA1057; 11, plasmid V517 derived from *E*. *coli* as the marker.

Southern hybridization analysis verified that the majority of *fosB* genes were on a small plasmid of about 2.5 kb (Fig 2). Seven of nine strains produced hybridization signal of *fosB* on one or two bands, and the other two strains (SA0620, SA1280) produced no hybridization signal.

### Conjugation Assay

Conjugation experiment verified that the plasmids harboring *fosB1*, *fosB4* or *fosB6* separately could confer fosfomycin resistance with the MIC ascending to 64 μg/ml. (Table 1).

### Multilocus Sequence Typing

The 9 *fosB* gene positive MRSA isolates were categorized into 4 ST types and same *fosB* subtype could be found in different ST strains (Table 1).

## Discussion

Nine of 67 strains in this study harbored *fosB* gene. Etienne *et al*. reported a 34% *fosB*-positive rate in 105 fosfomycin-resistant isolates of *Staphylococcus* spp. (18 *fosB*-positive strains in 39 *S*. *aureus* isolates) [8], which was a higher percentage of *fosB* positive isolates than we found. This may due to the diversity of strain origin or the larger number of *S*. *aureus* isolates examined in our study. Despite of a low detection rate of *fosB*, we unexpectively found three new subtypes of *fosB* gene, *fosB4*, *fosB5* and *fosB6*. The low homology between *fosB* and other *fos* genes were responsible for the different bacterial origins (Fig 1). On the other hand, the high homology between *fosB3* and other *fosB* subtypes implied a possible transfer between *Enterococcus faecium* and *Staphylococcus* spp. (Fig 1, Table 2).

The results of Southern hybridization analysis and conjugation assay show that the majority of *fosB* genes were on a small plasmid of about 2.5 kb (Fig 2). Some strains (SA1057, SA1159) with two *fosB* positive bands may be attributable to variations in the structure of the same plasmid (Fig 2). Two strains (SA0620, SA1280) produced no hybridization signal of *fosB*. This negative result might be attributed to a low copy number plasmid. By primer walking, we obtained two identical sequences adjacent to the *fosB5* genes from SA1280 and SA0620 and conjugation result suggested that they are more likely located on a larger plasmid.

Though whether *fosB5* gene is located on the plasmid or chromosome is not yet known clearly, we gain genetic environment of *fosB5* (Table 1 and Fig 2). Unlike the sequences flanking *fosB4* and *fosB6* which were similar to those found in *Staphylococcus* spp. [17–18], the sequences adjacent to *fosB5* gene has never been reported (Fig 1B). In addition to *rep* genes, there were two copies of the *tnp* gene with 99.4% nucleotide identity to IS*257* found in *S*. *aureus* [19]. The 17-bp sequence GGTTCTGTTGCAAAGTT of the terminal inverted repeat sequence (IR) exists at both ends of two copies of the IS*257*-like structure. Moreover, these IS*257*s share high identity in both their nucleotide and deduced amino acid sequences with the IS*15* family and IS*S1* founded in Gram-negative bacteria and *Streptococcus lactis*, respectively [20–22]. Plasmids harboring multiple copies of IS*257* may provide several sites for the excision or insertion of resistance genes through homologous recombination of an IS*257*-containing plasmid conferring erythromycin resistance (pOX7-IS) into the IS*257*s of pJ3356, as observed previously [19,23], implying that IS*257* is an active mobile genetic element conferring fosfomycin resistance.

Meanwhile, MLST profiles indicated that *fosB* genes were spreading in MRSA clinical strains. The result of conjugation also show that the *fosB* genes can be transferred and confer fosfomycin resistance to *S*. *aureus* ATCC25923. However, Although conjugants became resistant to fosfomycin after transformation with *fosB* genes, their MIC values were only 64 or 128 μg/ml, 2 to 3 times lower than observed in the donor strains. These results imply that other potential mechanisms contribute to fosfomycin resistance in MRSA.

In conclusion, we report three new *fosB* subtype genes that play a role in fosfomycin resistance in MRSA. Despite the diversity of these three *fosB* subtype genes in deduced amino acid and genetic environment, the strains bearing them could confer fosfomycin resistance by the plasmid or maybe through an active mobile genetic element. The different subtypes and genetic environment of *fosB* genes indicate that gene heterogeneity for fosfomycin resistance in MRSA isolates. Due to the complicated resistance and transmission mechanisms in fosfomycin-resistant MRSA, more research is warranted.
